# Direct comparison of current-induced spin polarization in topological insulator Bi_2_Se_3_ and InAs Rashba states

**DOI:** 10.1038/ncomms13518

**Published:** 2016-11-17

**Authors:** C. H. Li, O.M.J. van ‘t Erve, S. Rajput, L. Li, B. T. Jonker

**Affiliations:** 1 Materials Science and Technology Division, Naval Research Laboratory, Washington, District of Columbia 20375, USA; 2 Department of Physics, University of Wisconsin, Milwaukee, Wisconsin 53211, USA; 3 Department of Physics and Astronomy, West Virginia University, Morgantown, West Virginia 26506, USA

## Abstract

Three-dimensional topological insulators (TIs) exhibit time-reversal symmetry protected, linearly dispersing Dirac surface states with spin–momentum locking. Band bending at the TI surface may also lead to coexisting trivial two-dimensional electron gas (2DEG) states with parabolic energy dispersion. A bias current is expected to generate spin polarization in both systems, although with different magnitude and sign. Here we compare spin potentiometric measurements of bias current-generated spin polarization in Bi_2_Se_3_(111) where Dirac surface states coexist with trivial 2DEG states, and in InAs(001) where only trivial 2DEG states are present. We observe spin polarization arising from spin–momentum locking in both cases, with opposite signs of the measured spin voltage. We present a model based on spin dependent electrochemical potentials to directly derive the sign expected for the Dirac surface states, and show that the dominant contribution to the current-generated spin polarization in the TI is from the Dirac surface states.

## Abstract (web summary)

Spin-polarized states arising from either Rashba splitting or topological effects are expected to produce current-induced spin polarization with different magnitude and sign. Here, Li *et al*. observe current-generated spin polarization in both Bi_2_Se_3_ (111) and InAs (001) films, with opposite signs of the spin voltage.

## Graphical abstract

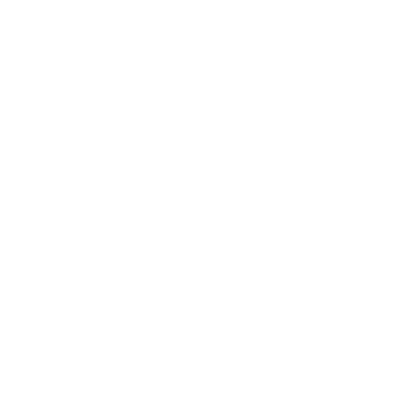

The quest for efficient generation and electrical control of spin has motivated the search for materials and structures that exhibit strong spin splitting of their electronic states^1^,^2^. A successful platform has been the two-dimensional electron gas (2DEG) in semiconductor heterostructures, where structural inversion asymmetry along surface normal lifts spin degeneracy via spin–orbit coupling^3^. In 2DEGs, with a parabolic energy dispersion, the Rashba form of spin–orbit coupling leads to a pair of Fermi surfaces that exhibit counter-rotating chiral spin texture, locking spin to the linear momentum^2^,^4^,^5^,^6^,^7^,^8^. The further demonstration of electrical gate control of the strength of such spin splitting has led to prospects for prototypical semiconductor field effect spintronic devices^8^,^9^.

Helical spin–momentum locking is also exhibited in the recently discovered quantum phase of matter, three-dimensional topological insulators (TIs), where linearly dispersing metallic surface states populated by massless Dirac fermions coexist with a semiconducting bulk^10^,^11^,^12^,^13^,^14^. The 2D Dirac states are occupied by a single spin, and topologically protected by time-reversal symmetry, making them robust against scattering. This helical spin texture has been observed by spin- and angle-resolved photoemission spectroscopy (ARPES)^15^,^16^,^17^. These measurements have also shown that trivial Rashba spin–split 2DEG states may coexist with the Dirac surface states in TI materials that exhibit surface carrier accumulation arising from band bending^18^,^19^.

An unpolarized bias current is predicted to create a net spin polarization due to spin–momentum locking for both the topologically protected TI surface states^20^,^21^,^22^ and the Rashba 2DEG states^3^,^4^. The spin helicities of the two have been shown in momentum-resolved measurements such as spin-ARPES to be opposite^18^. In transport measurements, however, the measured spin polarization is momentum integrated, and both Dirac and Rashba 2DEG states can contribute to the spin voltage measured at the detector contact. Calculations treating a model Bi_2_Se_3_ surface in which these states coexist found that the spin polarization and sign of the corresponding spin voltage measured at the detector contact is indeed opposite for the TI Dirac state and Rashba 2DEG contributions^23^,^24^. These calculations were performed for a three-terminal potentiometric geometry for both ballistic and diffusive regimes, using a spin–orbit coupling coefficient of *α*=0.79 Å obtained from ARPES measurements on Bi_2_Se_3_ (ref. 19). Furthermore, because the Rashba 2DEG states exists as spin–split pairs with Fermi level momenta **k**_1_ and **k**_2_, where **k**_1_*<***k**_2_, their spin contribution given by (**k**_2_–**k**_1_)/(**k**_2_+**k**_1_) mostly cancels, and the net spin polarization was shown to be dominated by the TI surface states^13^,^22^,^23^.

We recently demonstrated the first direct electrical detection of spin polarization resulting from spin–momentum locking in TI surface states in Bi_2_Se_3_ using spin potentiometric measurements, where the projection of the current-generated spin onto the magnetization of a ferromagnetic/tunnel barrier detector contact was measured as a voltage^25^. Similar investigations on various TI materials using similar FM/tunnel contacts^26^,^27^,^28^,^29^,^30^,^31^ have subsequently been reported. However, inconsistent results were reported regarding the sign of the spin signal, perhaps due to variations in material quality, device structure and measurement geometry, or the potential coexistence of the two spin systems. Lee *et al*.^29^ measure a spin–voltage consistent with ours^25^,^31^ in electrically gated (BiSb)_2_Te_3_ samples where the Fermi energy is systematically moved from the conduction band edge through the Dirac point to the valence band edge, while others report a spin voltage of opposite sign with a markedly different temperature dependence^28^. This underscores the need to independently probe and compare the characteristics of the Dirac and Rashba systems.

In this work, we report a direct comparison of the current-induced spin polarization measured using identical Fe/Al_2_O_3_ tunneling spin–potentiometric contacts and measurement geometries in two prototype systems: the TI Bi_2_Se_3_, where both Dirac surface states and Rashba 2DEG states are known to coexist; and InAs that exhibits only the Rashba 2DEG states. We show that the sign of the spin signal measured in the Bi_2_Se_3_ and InAs samples is indeed opposite, and the temperature dependence is markedly different. We further develop a model based on spin-dependent electrochemical potentials to explicitly illustrate the measurement and derive the sign of the spin voltage expected for the TI surface states, which corroborates our experimental observation here and in previous work^25^. These results show that the dominant contribution to the bias current-generated spin voltage in the TI is from the Dirac surface states in spin potentiometric measurements. This direct electrical access of the helical spin texture of Dirac and Rashba 2DEG states, along with recent demonstration of electrical-field-controlled magnetization switching in a magnetically doped TI^32^, highlight the exciting opportunities for future TI-based novel devices, such as the recently proposed TI/ferromagnet junction where transport can be altered from a spin-valve to an amplifier^33^.

## Results

### Potentiometric measurement of spin–momentum locking

**Figure 1:**
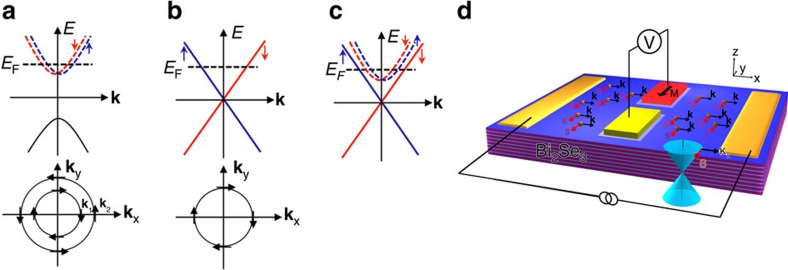
Spin–momentum locking and experimental concept. Schematic diagram of the spin–momentum locking textures of the Rashba 2DEG (**a**), Dirac surface states of TI (**b**), and coexistence of both (**c**). (**d**) Experimental concept of the potentiometric measurement.

A schematic diagram of the spin–momentum locking textures of the Rashba 2DEG is shown in Fig. 1a, where the Rashba form of spin–orbit coupling leads to a pair of Fermi surfaces with counter-rotating spins. For the Dirac surface states of TI (Fig. 1b), only one such Fermi surface exists and the 2D surface states are occupied by a single spin. For TI materials that exhibit surface carrier accumulation due to band bending, trivial Rashba spin–split 2DEG states can coexist with the TI Dirac surface states (Fig. 1c). The trivial 2DEG states are nested within the linear dispersing Dirac states^18^,^19^ such that the spin orientation of the higher **k** 2DEG state is opposite that of the nearest Dirac state. These spin textures arising from spin–momentum locking can be probed electrically using potentiometric measurements as shown in Fig. 1d, where the current-generated spin orientation is detected by its projection onto the ferromagnetic detector magnetization.

### Current-generated spin in TI Dirac states

**Figure 2:**
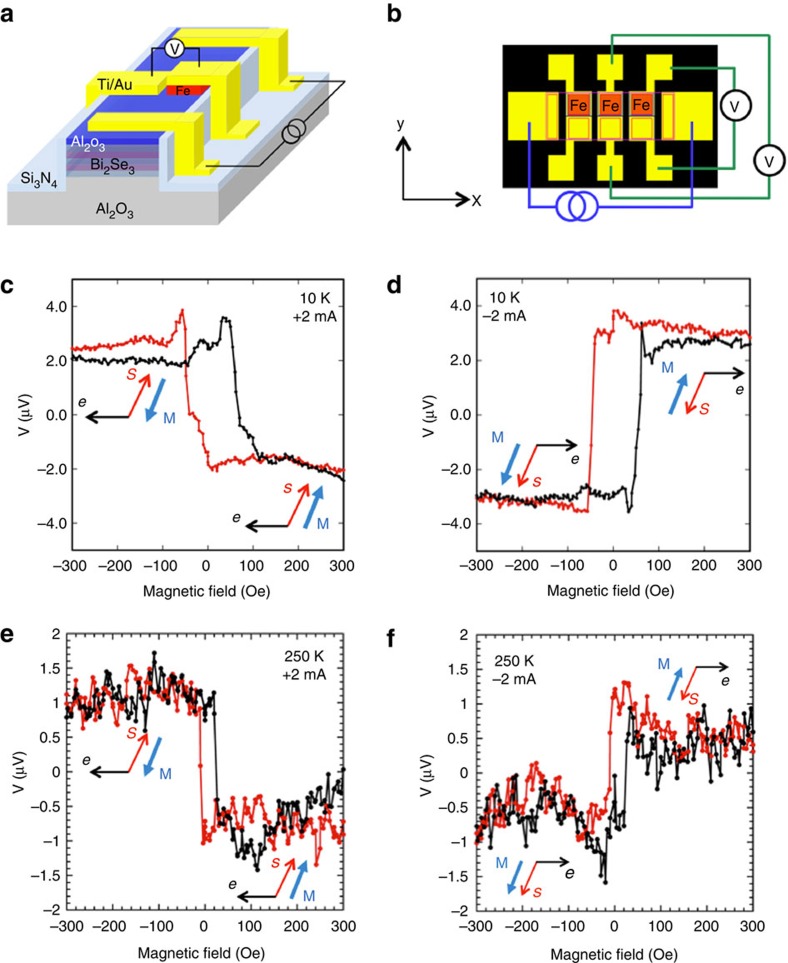
Device schematic and electrical detection of current-generated spin in TI. Schematic (**a**) and top view (**b**) of contact layout with two parallel rows of collinear detector contacts, top row is ferromagnetic (Fe, red), bottom row is non-magnetic reference (Ti/Au). Magnetic field dependence of the voltage measured at the ferromagnetic detector contact with the magnetization collinear with the induced TI spin for bias currents of +2 mA (**c**) and −2 mA (**d**). Similar measurements at 250 K at +2 mA (**e**) and −2 mA (**f**).

Bi_2_Se_3_ samples were grown by molecular beam epitaxy (MBE) on Al_2_O_3_(0001) substrates using a two-step process (see Methods). Structural and electronic properties of these films are provided in the Supplementary Figs 1–4. Fe/Al_2_O_3_ spin detector contacts were deposited on both Bi_2_Se_3_ and InAs samples in the same separate MBE system. The samples were then processed into identical device structures illustrated in Fig. 2a,b. Note that the geometry of the ferromagnetic detector contacts are specifically designed to cancel out the effect of fringe fields at the edges of the contacts (Supplementary Note 1). For the spin potentiometric measurements, a bias current is applied between the two Ti/Au current leads on either end of the device mesa, and a voltage is measured between the pairs of ferromagnetic (red) detector and corresponding non-magnetic Au/Ti (yellow) reference contacts.

When an unpolarized current flows between the two outer Ti/Au contacts, a spontaneous spin polarization is produced in the Bi_2_Se_3_ surface states throughout the channel due to spin–momentum locking. The projection of this spin onto the magnetization of the ferromagnetic detector contact is recorded as a voltage with a high-impedance voltmeter (>1 Giga-ohm). An in-plane magnetic field is applied to rotate the magnetization of the Fe detector contact, so that the projection of the current-generated spins onto the detector magnetization changes, resulting in a change in sign and magnitude of the detector voltage. Here we define the positive current to be holes flowing from left to right along the *+x* axis, and the positive magnetic field to be pointing in the *+y* direction.

The detector voltage as a function of magnetic field for +2 mA bias current at *T*=10 K is shown in Fig. 2c, after a simple linear background subtraction^25^ and centring around the vertical axis. Electron flow in the –*x* direction generates a spin in the +*y* direction due to spin–momentum locking in the TI Dirac states. At positive magnetic field>60 Oe, when the detector magnetization is saturated and completely parallel to the spin direction, a constant low voltage is observed. As the magnetic field decreases to small negative values around −50 Oe (coercive field of the magnetic contact; red trace), an abrupt increase in detector voltage is seen as the detector magnetization reverses to be antiparallel with the TI surface-state spin, and the overall scan exhibits a single step-like behaviour. When the field sweep direction is reversed to increase from negative to positive values (black trace), the detector voltage is constant until reaching the positive coercive field of 50 Oe, where the voltage abruptly decreases as the detector magnetization again switches and becomes parallel with the spin orientation of the TI surface state.

Changing the current direction to −2 mA (Fig. 2d), i.e., electrons flowing from left to right in the +x direction generate a spin in the –y direction. At positive magnetic field above saturation, the detector magnetization is antiparallel to the current-generated spin, and a constant high voltage is observed, while at negative magnetic field where the detector magnetization is parallel to the spin, a constant low voltage is seen. Comparing Fig. 2c,d, the hysteresis loop simply inverts around the *x* axis. This behaviour is very reproducible for temperatures up to 250 K, as is shown in Fig. 2e,f for both currents where clear hysteresis curves can still be seen.

### Temperature and bias dependence

**Figure 3:**
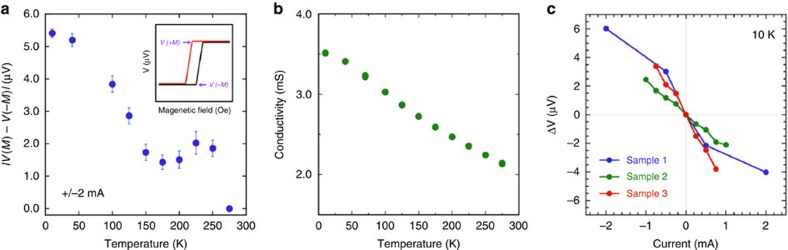
Temperature and bias dependence of TI spin voltage. (**a**) Temperature dependence of the spin voltage at +/−2 mA bias current. (Inset: illustration of how *ΔV=V(M)*−*V(*−*M)* is determined. Error bars are determined based on variations from a 10-point moving average.) (**b**) Temperature dependence of the conductivity. (**c**) Bias current dependence of the ferromagnetic detector voltage for several samples and devices showing a general linear dependence (Sample 1: 10 nm Fe/Al_2_O_3_/20 nm Bi_2_Se_3_/Al_2_O_3_, Sample 2, 3: 10 nm Fe/Al_2_O_3_/10 nm Bi_2_Se_3_/Al_2_O_3_).

The temperature dependence of the magnitude of the spin signal, |*V(+M)−V(−M)*|, measured at ±2 mA is shown in Fig. 3a. It decreases monotonically with increasing temperature to 175 K, exhibits a small increase between 175 and 250 K, and then disappears at 275 K. The small increase in the spin voltage in the range 175–250 K, and its abrupt suppression by 275 K are not well understood at present. This temperature dependence is similar to our previous observations of Bi_2_Se_3_ on graphene/n-SiC substrates^25^, although in our previous work the spin–voltage was observed only up to 150 K. The higher temperature achieved in this case for Bi_2_Se_3_ films grown directly on an insulating Al_2_O_3_ substrate could be attributed to the fact that there is no current shunting through the epi-graphene/SiC conductive substrate used previously, so that a higher fraction of the bias current flows in the TI surface layer to produce the measured spin polarization.

The temperature dependence of the spin signal is similar to that of the conductivity shown in Fig. 3b (which necessarily includes both surface state and bulk contributions), suggesting that both are reduced by scattering events as the temperature increases. These scattering events may arise from impurity scattering or interactions between surface state and bulk carriers^34^,^35^,^36^,^37^,^38^. For example, Dirac surface states can be scattered by impurities, leading to Dirac point fluctuations^34^, and phonons from the bulk states, which includes acoustic phonons that dominate at lower temperatures with a strong temperature dependence, as well as optical phonons that dominate at higher temperatures with a weaker temperature dependence^37^. Hybridization with bulk states can also lead to reduction of spin polarization^38^. In addition, the spin voltage may also be reduced by scattering between Rashba and Dirac states within the surface layer. Further experimental and theoretical work is required to understand such complex mechanisms and the resultant temperature dependence.

The dependence of the spin signal measured at the detector contact (*ΔV*=*V(+M)*−*V(*−*M)*) as a function of the bias current at *T*=10 K is shown in Fig. 3b, where a nearly linear dependence is observed. This linear behaviour of the spin signal with bias current is consistent with model calculations^23^, where the voltages measured on the FM detector *V(M)* were directly related to the bias current and spin polarization by [*V(+M)−V(−M)*]=*I*_*b*_*R*_*B*_*P*_*FM*_ (**ṗM**_u_), (bold case denotes a vector). Here *I*_*b*_ is the (hole) bias current in the +*x* direction, *R*_*B*_ is the ballistic resistance of the channel, and *P*_*FM*_ is the transport spin polarization of the FM detector metal. **M**_u_ is a unit vector along the detector magnetization **M**, and **p** is the degree of spin polarization induced per unit current by both spin–momentum locking in TI Dirac surface states and Rashba spin–orbit coupling in the 2DEG. From the spin signal we measure (for example, Fig. 3b), assuming that the bias current is shunted equally by each quintuple layer of the Bi_2_Se_3_ film^25^, and taking *P*_FM_ (Fe)∼0.4, and *k*_*F*_∼0.15 Å^−1^, we estimate **p**∼−0.15, with a sign that's indicative of the TI Dirac states^23^.

### Current-generated spin in InAs Rashba states

**Figure 4:**
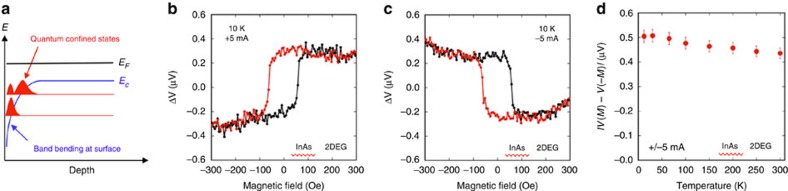
Electrical detection of current-generated spin in InAs 2DEG. (**a**) Schematic of InAs surface 2DEG formation. Magnetic field dependence of the voltage measured at the ferromagnetic detector contact with the magnetization collinear with the induced 2DEG spin in InAs for bias currents of +5 mA (**b**) and −5 mA (**c**). (**d**) Temperature dependence of the spin voltage at +/−5 mA bias current. (Error bars are determined based on variations from a 10 point moving average.)

To further distinguish the sign of the spin signal measured for the TI Dirac surface states from that of potential trivial 2DEG states, we performed similar measurements on InAs(001) samples where only the surface 2DEG states are known to exist^39^,^40^,^41^,^42^,^43^. It is well known that the downward band bending of the conduction band at the InAs(001) surface leads to an electron accumulation layer and the formation of a surface 2DEG^39^,^40^,^41^,^42^,^43^ (Fig. 4a) that extends ∼20 nm into the sample^43^. The InAs samples were processed to produce the same Fe/Al_2_O_3_ contact geometry used for the Bi_2_Se_3_ measurements (Materials and Methods). As noted earlier, the Rashba spin–orbit-induced polarization is predicted to exhibit the opposite sign to that of TI Dirac states^23^ for a given bias current.

The spin–voltage transport data for these InAs samples are shown in Fig. 4b,c. The measurement procedures were identical to those used for the Bi_2_Se_3_ samples. Similar hysteresis loops are observed where a constant high and low voltage is measured when the detector magnetization is fully aligned with the applied field. However, for a given current/electron flow direction, the hysteresis loop is clearly inverted about the horizontal axis relative to that observed for the Bi_2_Se_3_ samples (Fig. 2c–f): for a positive bias current, a high-voltage signal is seen at positive fields above the coercive field of the Fe contact, and a low voltage is observed at negative fields. Given that the spin-detecting contacts (Fe/Al_2_O_3_) and contact geometry are the same, and are sensitive only to the orientation of the induced spin polarization regardless of the source, this observation indicates that the bias current-induced spin polarization due to spin–momentum locking in the InAs 2DEG is opposite to that of the Bi_2_Se_3_ TI Dirac states, consistent with the theory^23^. In addition, the spin voltage exhibits a weak temperature dependence, decreasing by only ∼10% from 10 to 300 K, and persists to at least 300 K, as shown in Fig. 4d, consistent with the metallic nature of the 2DEG. This is markedly different than the strong temperature dependence observed for the TI Dirac surface state (Fig. 3a).

## Discussion

While both the topologically protected Dirac surface states and the Rashba spin–split 2DEG states of the Bi_2_Se_3_ are expected to produce a bias current induced spin polarization due to spin–momentum locking, the current-induced spin density is expected to be substantially larger for the Dirac surface states for several reasons. First, as discussed above, for a given momentum direction, the Rashba 2DEG states exist as spin–split pairs of opposite spin orientation, and the net induced spin polarization is proportional to (**k**_2_−**k**_1_)/(**k**_2_+**k**_1_) (ref. 23), where **k**_2_>**k**_1_ (Fig. 1a). Consequently, the contributions from these spin–split states tend to cancel. In contrast, the Dirac state has only one spin orientation, and no such cancellation occurs. Second, the induced spin polarization is enhanced by a factor *v*_*F*_*/α*>>1 for the TI Dirac surface states, where *v*_*F*_ is the Fermi velocity of the TI (on the order of 10^5^ m s^−1^) and *α* the strength of the Rashba spin–orbit coefficient in the 2DEG in units of velocity (on the order of 10^3^ m s^−1^)^13^,^20^,^22^. The fact that in degenerate Bi_2_Se_3_ samples where both the Dirac and Rashba states coexist, the sign of the spin signal we observe corresponds to that of the TI Dirac states, corroborates the prediction and expectation that the signal should be dominated by contributions from the Dirac states.

As noted earlier, there are inconsistencies in the sign of the spin signal [*V(+M)*−*V(*−*M)*] reported for nominally identical measurements of the bias current-induced spin polarization attributed to the TI Dirac states, even for the same Bi_2_Se_3_ material (and stated conventions for current and magnetic field directions). This is reflected in whether a low- or high-voltage signal is observed when the detector magnetization is parallel or antiparallel to the induced spin. In our measurements of the TI Dirac surface states, we observe [*V(+M)*−*V(*−*M)*]<0, that is, a low-voltage signal when the detector magnetization and TI spin are parallel, and a high signal when antiparallel (Fig. 2; ref. 25), as have other groups^29^. In contrast, others report the opposite behaviour^28^,^30^, as well as markedly different temperature dependence for the spin voltage in Bi_2_Se_3_ films^30^.

Hence, to directly derive the sign of the spin voltage that should be expected, we develop a model based on the spin-dependent electrochemical potentials generated and their detection by a ferromagnetic detector contact. We note that similar models have been reported in refs 28, 30, although the gradients and/or the reference of the electrochemical potentials are inconsistent with conventional usage in the spintronics community. To better illustrate and contrast the discrepancies, we construct our diagram using notation similar to that of ref. 28.

**Figure 5:**
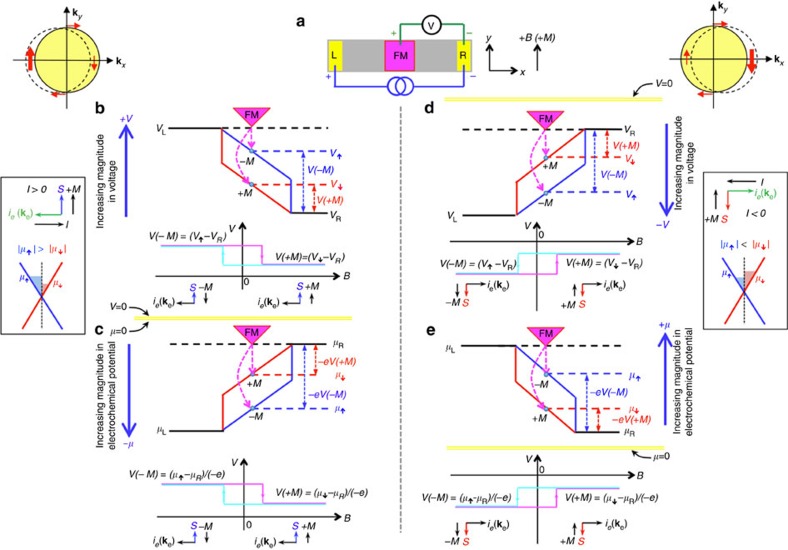
Model to derive sign of spin signal expected in TI. (**a**) Schematic of a simplified 3-terminal device with a left (L) and right (R) electrode and central magnetic detector contact (FM), as well as definitions of voltage (*V*) terminals, magnetic field (*B*) and magnetization (*M*) directions. Model of the spin potentiometric measurement probing the current-induced spin polarization due to TI surface states, based on the spin-dependent electrochemical potentials: for positive bias currents using voltage profiles (**b**) and electrochemical potential (*μ*) profiles (**c**), and for negative bias currents in the form of voltage profiles (**d**) and electrochemical potential profiles (**e**). The definitions of current, spin (*S*), and magnetization directions are indicated for positive currents next to **b**,**c**, and for negative currents next to **d**,**e**. The blue and red solid lines indicate profiles for the spin-up (↑) and spin-down (↓) electrons, respectively. The blue and red horizontal dashed lines indicate the levels been probed by corresponding magnetization directions. For example, for **b**,**c**, the spin-up (blue) voltage and electrochemical potentials are probed by the FM detector with –*M* magnetization (oriented along −*y*), which has its majority spin oriented along +*y* (due to the opposite alignment of majority spin to its magnetization in magnetic metals^47^). The yellow horizontal lines indicate the zero axis for the voltage and electrochemical potential (*V*=*μ*=0), which is shared for **b**,**c**, and individually marked for **d**,**e**.

We begin with a simple 3-terminal measurement geometry similar to that of Hong *et al*.^23^ shown in Fig. 5a. We define the left contact as the positive terminal, and the right contact as the negative terminal, or the reference contact, as used in our measurements. The positive magnetic field direction (and detector magnetization) is again defined to be in the +*y* direction, with positive (hole) current flowing in the +*x* direction. We present a diagram of the electric field and voltage (*V*), where *V* is directly related to electrochemical potential (*μ*) by *μ=*−*eV* (refs 44, 45), where *e* is the electron charge (taken to be a positive quantity). With these conventions, the voltage reference point and gradient of the electric field are unambiguously defined. In the following, we discuss the measurement in terms of both the voltage and electrochemical potential.

For a positive current (*I>0*; Fig. 5b,c), electron momentum **k**_e_ is from right to left in the *–x* direction, and the voltage of the left electrode (*V*_L_) is high relative to that of the right electrode (*V*_R_). The right (reference) contact need not be grounded, so we indicate a zero reference *V*=*μ*=0 by the yellow line common to Fig. 5b,c. Thus, the gradient of the electric field has a negative slope (Fig. 5b). The profile of the electrochemical potential (Fig. 5c), *μ=*−*eV*, is merely the mirror image of the electric field/voltage profile across the *V*=*μ*=0 axis. Here the left electrode (L) has a more negative (larger magnitude) electrochemical potential than the right electrode (R), and the profile has a positive slope.

When a bias current flows through the TI Dirac surface states, a net spin polarization is created due to spin–momentum locking with a direction determined by the electron momentum: for **k**_e_ along the –*x* direction (positive bias current), the induced spin is oriented along the +*y* axis and referred to as spin-up. Consequently, the electrochemical potential splits for spin-up and spin-down electrons, as represented by the blue *μ*_↑_ and red *μ*_↓_ lines in Fig. 5c, where *μ*_↑_ for the spin-up electrons is larger (in magnitude, that is, more negative) than that of the spin-down *μ*_↓_ (i.e., |*μ*_↑_|>|*μ*_↓_|). The corresponding levels in the voltage diagram of Fig. 5b are again the mirror image across the horizontal axis.

This spin imbalance is probed by the ferromagnetic detector contact. The magnetization of the ferromagnet aligns with the applied external magnetic field above saturation. However, its magnetic moment is opposite to the orientation of its majority spin^46^. Hence, the FM detector with *+M* magnetization (oriented along *+y*) has its majority spin oriented along *–y*, and will probe the spin-down electrochemical potential (*μ*_↓_, *V*_↓_) in the channel. Conversely, the detector with −*M* magnetization probes the spin-up levels (*μ*_↑_, *V*_↑_).

Since the right electrode (R) is the reference, when the detector magnetization is saturated at positive magnetic field, its voltage *V(+M*) due to probing the spin-down electron band is −*eV(+M)*=*μ*_↓_−*μ*_R_, or *V(+M)=(μ*_↓_−*μ*_R_*)/(*−*e)*. Similarly, when the detector magnetization is saturated at negative magnetic field, its voltage due to probing the spin-up band is *V(−M)* =(*μ*_↑_−*μ*_R_*)/(*−*e)*. Since |*μ*_↑_|>|*μ*_↓_|, this yields a high voltage signal at the negative field, when the magnetization is antiparallel to the TI spin (spin-up), and a low voltage at positive field, when it is parallel to the TI spin, as depicted by the hysteresis loop below the potential diagram in Fig. 5c. Note that a simple linear background subtraction and centring around the vertical axis does not change the relative high and low signals.

A similar analysis can be made directly in terms of the voltage, as shown in the top panels of Fig. 5b. Electrons flowing from right to left in the –*x* direction create a net spin-up population oriented along *+y*, and hence the blue *V*_↑_ level is higher (that is, larger in magnitude) than that of the spin-down *V*_↓_ (that is, *V*_↑_>*V*_↓_), analogous to |*μ*_↑_|>|*μ*_↓_|. With the right electrode (R) as the reference, the detector voltage at positive magnetic field (*+B, +M*) probing the spin-down level is *V(+M)=(V*_↓_−*V*_R_), and that at negative magnetic field (−*B*, −*M*) which probes spin-up is *V(*−*M)=(V*_↑_−*V*_R_). And since *V*_↑_>*V*_↓_, at positive field (when magnetization is parallel to TI spin), a low voltage is expected, and at negative field (magnetization antiparallel to TI spin), a high voltage is expected (or *ΔV* (=*V(+M)*−*V(*−*M))* should be negative), exactly as we observe experimentally in Bi_2_Se_3_.

This exercise can be repeated when the current direction (and hence induced spin direction) is reversed, as shown in Fig. 5d,e. We note that discrepancies/mistakes can be made when discussing electrochemical potentials with negative values^28^,^30^. However, these can be avoided by establishing the voltage potential first, as derived above. Another potential complication is that the measured spin voltage from the trivial 2DEG states is also sensitive to the sign and value of the Rashba spin–orbit coupling parameter *α* (ref. 23), which can vary depending on the nature of the interface^47^. In the current case, a positive value of α has been reported for various types of TI and InAs in the literature, hence no sign reversal should be expected than that predicted in refs 23, 24.

In summary, we directly compare electrical measurements of current-generated spin polarization due to spin–momentum locking in two complimentary systems: Bi_2_Se_3_ with the potential coexistence of both Dirac and trivial Rashba surface states, and InAs with only the Rashba states. We show that the spin voltages measured for the Dirac and Rashba systems are indeed opposite in sign, as predicted by theory^23^. We further develop a model based on spin-splitting of the electrochemical potential to derive the sign of the spin voltage expected for the Dirac states from a potentiometric measurement using a ferromagnetic contact, further confirming that the spin signals we observe from the Bi_2_Se_3_ are consistent with the Dirac surface states. These results demonstrate that the current-generated spin polarization in non-trivial TI Dirac and trivial Rashba 2DEG states are indeed opposite, as expected from their different energy band dispersion, and that in a TI it is dominated by the Dirac surface states. These demonstrations of direct electrical detection of the helical spin texture of Dirac and Rashba states is an enabling step towards electrical manipulation of spins in future TI-based quantum devices.

## Methods

### Sample growth

The growth of Bi_2_Se_3_ films was carried out on Al_2_O_3_(0001) substrates in an ultrahigh vacuum (UHV) system (base pressure ∼1 × 10^−10^ torr) that integrates two MBE chambers and a low temperature (500–300 K) scanning tunneling microscope. For the growth of Bi_2_Se_3_, a two-step process is used^48^: 2–3 quintuple layers (QL) of Bi_2_Se_3_ were first deposited at a reduced temperature of 100 °C, and the substrate temperature was then slowly raised to 300 °C where the rest of the film was deposited. Bi and Se were supplied via separate Knudsen cells at 460 and 250 °C, respectively^49^. The samples were then removed to air and transferred to a separate MBE system where Fe/Al_2_O_3_ contacts were deposited as described previously^50^ and below.

In the case of InAs, an undoped InAs(001) substrate was heated to 520 °C in an As flux to desorb the oxide. The sample was then cooled to room temperature and transferred under ultrahigh vacuum into an interconnected MBE system for the growth of Fe/Al_2_O_3_ (the same system used to deposit Fe/Al_2_O_3_ on Bi_2_Se_3_).

The Fe/Al_2_O_3_ contacts were formed on Bi_2_Se_3_ as follows. A 0.7 nm layer of polycrystalline Al was first deposited by MBE, and then oxidized in 200 torr O_2_ for 20 min in the presence of ultraviolet light in the load-lock chamber. This step was then repeated for a total Al_2_O_3_ thickness of 2 nm. The sample was then transferred under UHV to an interconnected metals MBE chamber, where 20 nm of polycrystalline Fe was deposited at room temperature from a Knudsen cell.

### Sample processing

The samples were processed into the device structures illustrated in Fig. 2a,b to enable transport measurements. Standard photolithography and chemical etching methods were used to define the Fe contacts, which ranged in size from 20 × 20 μm^2^ to 80 × 80 μm^2^, with adjacent contact separation ranging from 45 to 200 μm. Ion milling was used to pattern the Bi_2_Se_3_ mesa. Large Ti/Au contacts were deposited by lift-off in an electron beam evaporator as non-magnetic reference contacts and bias current leads. The Fe contacts were capped with 10 nm Ti/100 nm Au, and bond pads for wire bonded electrical connections are further electrically isolated using 100 nm of Si_3_N_4_.

### Transport measurements

Transport measurements were performed in a closed cycle cryostat equipped with an electromagnet (4–300 K, ±1,000 Oe). An unpolarized bias current was applied through the outer Ti/Au contacts on the opposite ends of the Bi_2_Se_3_ mesa, and the voltage on the detector contact was recorded as a function of the in-plane magnetic field applied orthogonal to the electron bias current direction in the TI.

### Materials characterization

Topological insulator thin films are grown by molecular beam epitaxy and characterized by a variety of techniques. Synchrotron-based high-resolution X-ray Reflectivity was carried out at the Argonne National Lab in collaboration with Dr Jonathan Emery of Northwestern University. Raman spectroscopy was done at the University of Wisconsin, Milwaukee. High resolution transmission electron microscopy and associated high angle annular dark field imaging was done at the University of York, UK in collaboration with Dr Vlado Lazarov. Scanning tunneling microscopy and spectroscopy was carried out *in situ* after growth at 77 K at University of Wisconsin, Milwaukee.

### Data availability

The data sets generated during and/or analysed during the current study are available from the corresponding author on reasonable request.

## Additional information

**How to cite this article:** Li, C. H. *et al*. Direct comparison of current-induced spin polarization in topological insulator Bi_2_Se_3_ and InAs Rashba states. *Nat. Commun.* **7,** 13518 doi: 10.1038/ncomms13518 (2016).

**Publisher's note:** Springer Nature remains neutral with regard to jurisdictional claims in published maps and institutional affiliations.

## Supplementary Material

Supplementary Information Supplementary Figures 1-5, Supplementary Note 1 and Supplementary References.

## References

[b1] Murakawa H. . Detection of Berry's Phase in a Bulk Rashba Semiconductor. Science 342, 1490–1493 (2013). 24357313 10.1126/science.1242247

[b2] Manchon A., Koo H. C., Nitta J., Frolov S. M. & Duine R. A. New perspectives for Rashba spin-orbit coupling. Nat. Mater. 14, 871 (2015). 26288976 10.1038/nmat4360

[b3] Bychkov Y. A. & Rashba E. I. Properties of a 2D electron gas with lifted spectral degeneracy. JEPT Lett. 39, 78 (1984).

[b4] Vas'ko F. T. Spin splitting in the spectrum of two-dimensional electrons due to the surface potential. P. Zh. Eksp. Teor. Fiz. 30, 574–577 (1979).

[b5] Hammar P. R. & Johnson M. Potentiometric measurements of the spin-split subbands in a two-dimensional electron gas. Phys. Rev. B 61, 7207 (2000).

[b6] Silsbee R. H. Theory of the detection of current-induced spin polarization in a two-dimensional electron gas. Phys. Rev. B 63, 155305 (2001).

[b7] Nitta J., Akazaki T., Takayanagi H. & Enoki T. Gate control of spin-orbit interaction in an inverted In_0.53_Ga_0.47_As/In_0.52_Al_0.48_As heterostructure. Phys. Rev. Lett. 77, 3417 (1996).

[b8] Koo H. C. . Control of spin precession in a spin-injected field effect transistor. Science 325, 1515 (2009). 19762637 10.1126/science.1173667

[b9] Datta S. & Das B. Electronic analog of the electro-optic modulator. Appl. Phys. Lett. 56, 665 (1990).

[b10] Moore J. E. The birth of topological insulators. Nature 464, 194–198 (2010). 20220837 10.1038/nature08916

[b11] Hasan M. Z. & Kane C. L. Colloquium: topological insulators. Rev. Mod. Phys. 82, 3045–3067 (2010).

[b12] Fu L., Kane C. L. & Mele E. J. Topological insulators in three dimensions. Phys. Rev. Lett. 98, 106803 (2007). 17358555 10.1103/PhysRevLett.98.106803

[b13] Pesin D. & MacDonald A. H. Spintronics and pseudospintronics in graphene and topological insulators. Nat. Mater. 11, 409–416 (2012). 22522641 10.1038/nmat3305

[b14] Kong D. & Cui Y. Opportunities in chemistry and materials science for topological insulators and their nanostructures. Nat. Chem. 3, 845–849 (2011). 22024879 10.1038/nchem.1171

[b15] Hsieh D. . A topological Dirac insulator in a quantum spin Hall phase. Nature 452, 970 (2008). 18432240 10.1038/nature06843

[b16] Zhang H. . Topological insulators in Bi_2_Se_3_, Bi_2_Te_3_ and Sb_2_Te_3_ with a single Dirac cone on the surface. Nat. Phys. 5, 438–442 (2009).

[b17] Hsieh D. . A tunable topological insulator in the spin helical Dirac transport regime. Nature 460, 1101–1105 (2009). 19620959 10.1038/nature08234

[b18] Bahramy M. S. . Emergent quantum confinement at topological insulator surfaces. Nat. Commun. 3, 1159 (2012). 23093196 10.1038/ncomms2162

[b19] King P. D. C. . Large tunable rashba spin splitting of a two-dimensional electron gas in Bi_2_Se_3_. Phys. Rev. Lett. 107, 096802 (2011). 21929260 10.1103/PhysRevLett.107.096802

[b20] Burkov A. A. & Hawthorn D. G. Spin and charge transport on the surface of a topological insulator. Phys. Rev. Lett. 105, 066802 (2010). 20867997 10.1103/PhysRevLett.105.066802

[b21] Culcer D., Hwang E. H., Stanescu T. D. & Das Sarma S. Two-dimensional surface charge transport in topological insulators. Phys. Rev. B 82, 155457 (2010).

[b22] Yazyev V., Moore J. E. & Louie S. G. Spin polarization and transport of surface states in the topological insulators Bi_2_Se_3_ and Bi_2_Te_3_ from first principles. Phys. Rev. Lett. 105, 266806 (2010). 21231702 10.1103/PhysRevLett.105.266806

[b23] Hong S., Diep V., Datta S. & Chen Y. P. Modeling potentiometric measurements in topological insulators including parallel channels. Phys. Rev. B. 86, 085131 (2012).

[b24] Hong S., Sayed S. & Datta S. Spin circuit model for 2D channels with spin-orbit coupling. Sci. Rep. 6, 20325 (2016). 26932563 10.1038/srep20325 PMC4773926

[b25] Li C. H. . Electrical detection of charge-current-induced spin polarization due to spin-momentum locking in Bi_2_Se_3_. Nat. Nanotechnol. 9, 218–224 (2014). 24561354 10.1038/nnano.2014.16

[b26] Tang J. . Electrical detection of spin-polarized surface states conduction in (Bi_0.53_Sb_0.47_)_2_Te_3_ topological insulator. Nano Lett. 14, 5423–5429 (2014). 25158276 10.1021/nl5026198

[b27] Ando Y. . Electrical detection of the spin polarization due to charge flow in the surface state of the topological insulator Bi_1.5_Sb_0.5_Te_1.7_Se_1.3_. Nano Lett. 14, 6226–6230 (2014). 25330016 10.1021/nl502546c

[b28] Tian J., Miotkowski I., Hong S. & Chen Y. P. Electrical injection and detection of spin-polarized currents in topological insulator Bi_2_Te_2_Se. Sci. Rep. 5, 14293 (2015). 26391089 10.1038/srep14293 PMC4585757

[b29] Lee J. S., Richardella A., Hickey D. R., Mkhoyan K. A. & Samarth N. Mapping the chemical potential dependence of current-induced spin polarization in a topological insulator. Phys. Rev. B 92, 155312 (2015).

[b30] Dankert A., Geurs J., Kamalakar M. V. & Dash S. P. Room temperature electrical detection of spin polarized currents in topological insulators. Nano Lett. 15, 7976–7981 (2015). 26560203 10.1021/acs.nanolett.5b03080

[b31] Li C. H. . Electrical detection of the helical spin texture in a p-type topological insulator Sb_2_Te_3_. Sci. Rep. 6, 29533 (2016). 27404321 10.1038/srep29533 PMC4941728

[b32] Fan Y. . Electric-field control of spin–orbit torque in a magnetically doped topological insulator. Nat. Nanotechnol. 11, 352–359 (2016). 26727198 10.1038/nnano.2015.294

[b33] Scharf B., Matos-Abiague A., Han J. E., Hankiewicz E. M. & Žutić I. Tunneling planar hall effect in topological insulators: spin-valves and amplifiers. Phys. Rev. Lett. 117, 166806–2016. 10.1103/PhysRevLett.117.166806 27792378

[b34] Beidenkopf H. . Spatial fluctuations of helical Dirac fermions on the surface of topological insulators. Nat. Phys. 7, 939–943 (2011).

[b35] Kim S. . Surface scattering via bulk continuum states in the 3D topological insulator Bi_2_Se_3_. Phys. Rev. Lett. 107, 56803 (2011). 10.1103/PhysRevLett.107.056803 21867088

[b36] Sessi P. . Visualizing spin-dependent bulk scattering and breakdown of the linear dispersion relation in Bi_2_Te_3_. Phys. Rev. B 88, 161407 (2013).

[b37] Saha K. & Garate I. Theory of bulk-surface coupling in topological insulator films. Phys. Rev. B 90, 245418 (2014).

[b38] Hsu Y.-T., Fischer M. H., Hughes T. L., Park K. & Kim E.-A. Effects of surface-bulk hybridization in three-dimensional topological metals. Phys. Rev. B 89, 205438 (2014).

[b39] Wieder H. H. Transport coefficients of InAs epilayers. Appl. Phys. Lett. 25, 206–208 (1974).

[b40] Tsui D. C. Observation of surface bound state and two-dimensional energy band by electron tunneling. Phys. Rev. Lett. 24, 303 (1970).

[b41] Tsui D. C. Landau-level spectra of conduction electrons at an InAs surface. Phys. Rev. B 12, 5739–5748 (1975).

[b42] Wang P. D. . Electrical and magneto-optical studies of MBE InAs on GaAs. Semicond. Sci. Technol. 7, 767–786 (1992).

[b43] Wolkenberg A. & Przeslawski T. Charge transport diagnosis by: I-V (resistivity), screening and Debye length, mean free path, Mott effect and Bohr radius in InAs, In_0.53_Ga_0.47_As and GaAs MBE epitaxial layers. Appl. Surf. Sci. 254, 6736–6741 (2008).

[b44] Van Son P. C., van Kempen H. & Wyder P. Boundary Resistance of the Ferromagnetic-Nonferromagnetic Metal Interface. Phys. Rev. Lett. 58, 2271–2273 (1987). 10034698 10.1103/PhysRevLett.58.2271

[b45] Datta S. Quantum Transport: Atom to Transistor Cambridge Univ. Press (2005).

[b46] Jonker B. T., Hanbicki A. T., Pierce D. T. & Stiles M. D. Spin nomenclature for semiconductors and magnetic metals. J. Magn. Magn. Mater. 277, 24 (2004).

[b47] Gmitra M., Matos-Abiague A., Draxl C. & Fabian J. Magnetic control of spin-orbit fields: a first-principles study of Fe/GaAs junctions. Phys. Rev. Lett. 111, 036603 (2016). 10.1103/PhysRevLett.111.036603 23909348

[b48] Li H. D. . The van der Waals epitaxy of Bi_2_Se_3_ on the vicinal Si(111) surface: an approach for preparing high-quality thin films of a topological insulator. New J. Phys. 12, 103038 (2010).

[b49] Liu Y., Weinert M. & Li L. Spiral growth without dislocations: molecular beam epitaxy of the topological insulator Bi_2_Se_3_ on epitaxial graphene/SiC(0001). Phys. Rev. Lett. 108, 115501 (2012). 22540484 10.1103/PhysRevLett.108.115501

[b50] Jonker B. T., Kioseoglou G., Hanbicki A. T., Li C. H. & Thompson P. E. ‘Electrical spin injection into silicon from a ferromagnetic metal/tunnel barrier contact'. Nat. Phys. 3, 542 (2007).

