# Supplementary material for: Direct comparison of current-induced spin polarization in topological insulator Bi2Se3 and InAs Rashba states
Source: Nat Commun. 2016 Nov 17;7:13518. doi: 10.1038/ncomms13518 (PMC5118552; doi:10.1038/ncomms13518)
Supplement: Supplementary Information — Supplementary Figures 1-5, Supplementary Note 1 and Supplementary References. [file ncomms13518-s1.pdf]

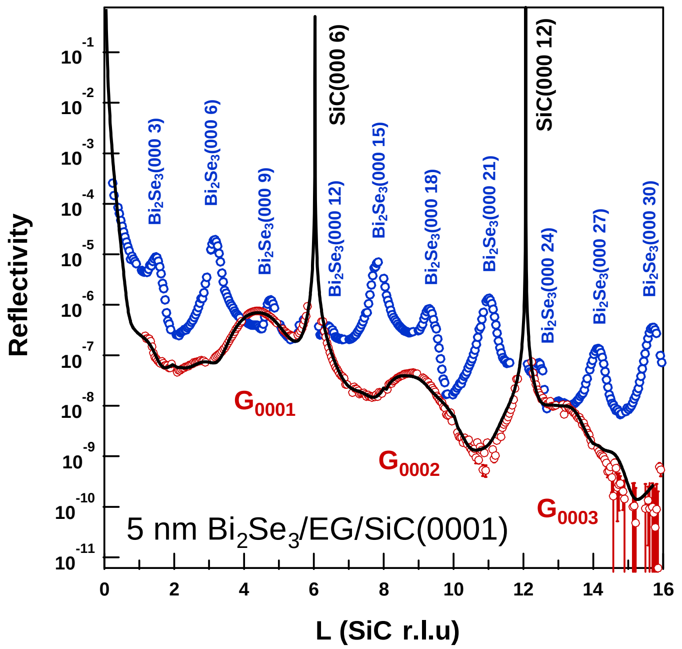

Supplementary Figure 1 **Structural characterization by x-ray reflectivity.** High-resolution X-ray Reflectivity spectra of a Bi_2Se_3 thin film grown on epitaxial graphene (EG)/SiC(0001), where only reflections from the 003 family of planes are seen, including many higher order peaks, indicating a highly c-axis oriented film along the growth direction, and the high quality of the film.

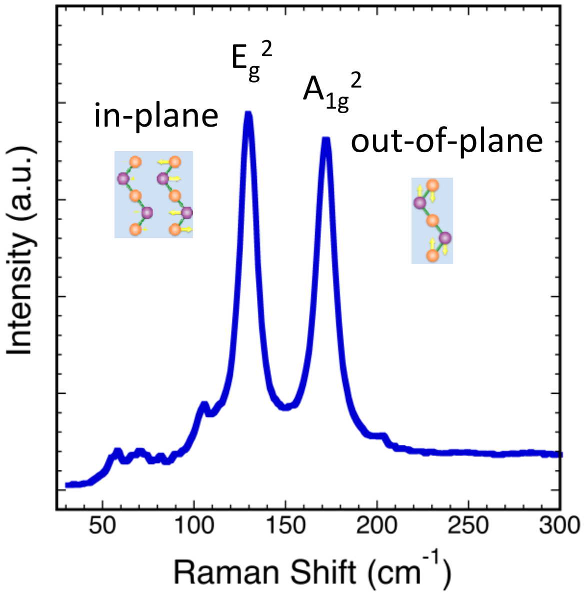

Supplementary Figure 2 **Raman characterization.** Raman spectrum of a Bi_2Se_3 film showing the characteristic in-plane E_g^2 and out of plane A_{1g}^2 vibrational modes.

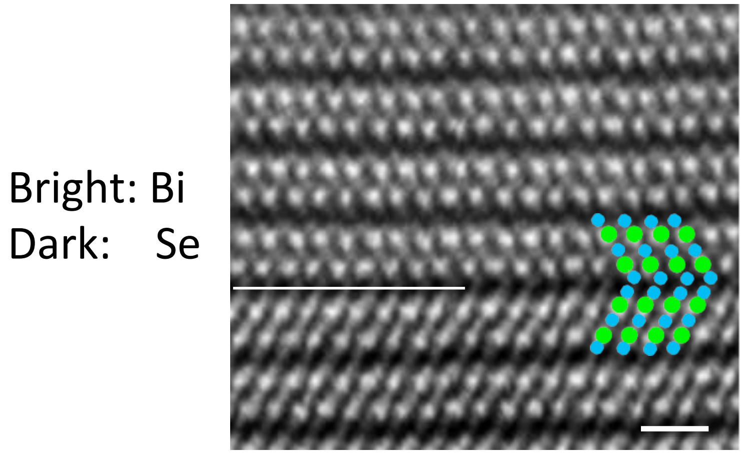

Supplementary Figure 3 **Structural characterization by transmission electron microscopy.** Z-contrast High Angle Annular Dark Field (HAADF) image of the cross section of a Bi_2Se_3 film showing good crystalline quality and well-ordered structure. The darker atoms are Se and brighter ones Bi. (Scale bar: 1 nm)

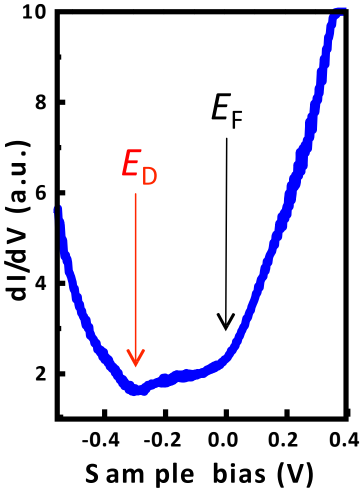

Supplementary Figure 4 **In situ tunneling spectrum.** Scanning tunneling spectrum on Bi_2Se_3 grown on epitaxial graphene/SiC taken at 77 K. The Dirac point (E_D, pointed by red arrow) is clearly present, at ~ 300 meV below the Fermi level (E_F) indicate n-type doping.

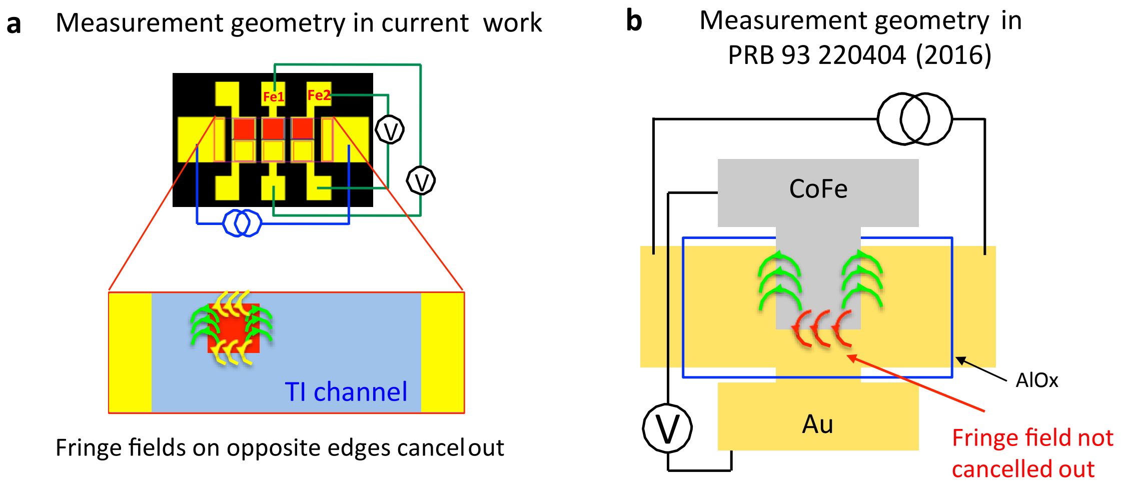

Supplementary Figure 5 **Fringe fields at edges of magnetic detector contacts.** (a) Fringe fields are cancelled out in the measurement geometry of current study. (b) Fringe fields are not cancelled out in the measurement geometry shown in Ref. 1 (b).

Supplementary Note 1: Fringe field at edges of magnetic detector contacts.

The spin potentiometric measurements of bias current-generated spin polarization requires the ferromagnetic (FM) contacts to be carefully designed such that artifacts such as those arising from fringe fields at the edges of the contacts do not contribute to the spin signal. As shown below in the left panel, our FM contacts are square where all 4 edges are within the width of the channel, where the fringe field on opposite sides are opposite in direction and should cancel out.

In contrast, in a recently published work [1], the authors show a similar hysteric behavior on the magnetic CoFe detector contact with a current flowing through Au. We note that the measurement geometry used in this study (c.f. Fig. 2 inset (a) in Ref. 1) does not preclude the uneven fringe field from the edge of the magnetic contact giving rise to the observed signal. In fact, a similar geometry was used by Johnson et al. [2] where the fringe field is utilized to manipulate current flow in an InAs 2DEG to purposefully produce a maximum Hall voltage.

Supplementary References

1. Li, P., Appelbaum, I., Interpreting current-induced spin polarization in topological insulator surface states. *Phys. Rev. B* **93**, 220404 (2016).
2. Johnson, M., Hybrid ferromagnet–semiconductor devices. *J. Vac. Sci. Technol. A* **16,** 1806-1811 (1998).
